# Supplementary material for: Handling missing modalities in multimodal survival prediction for non-small cell lung cancer
Source: NPJ Digit Med. 2026 Jun 22;9:776. doi: 10.1038/s41746-026-02783-3 (PMC13649774; doi:10.1038/s41746-026-02783-3)
Supplement: Supplementary file 1 [file 41746_2026_2783_MOESM1_ESM.pdf]

Supplementary Materials

S.1 Ablation Analysis on Multimodal Module Design

Layer 1	Layer 2	MS	CT+Tab	WSI+CT	WSI+CT+Tab	WSI+Tab
FC	FC	Un-frozen	68.52 ± 5.41	72.31 ± 4.76	73.49 ± 4.55	69.39 ± 2.90
		Frozen	68.94 ± 5.88	**76.12** ± 4.01	70.85 ± 3.66	67.54 ± 4.33
FC	ODST	Un-frozen	**69.90** ± 5.08	72.49 ± 3.50	73.09 ± 3.79	**73.30** ± 3.23
		Frozen	**69.90** ± 4.94	73.38 ± 3.67	**74.42** ± 4.25	70.63 ± 1.41
ODST	FC	Un-frozen	69.18 ± 4.79	73.38 ± 4.35	73.38 ± 3.66	67.48 ± 2.79
		Frozen	68.78 ± 5.47	72.75 ± 4.50	74.02 ± 3.61	68.00 ± 1.80
ODST	ODST	Un-frozen	69.18 ± 4.79	72.97 ± 4.11	72.92 ± 4.86	68.13 ± 3.59
		Frozen	69.26 ± 5.32	72.56 ± 3.60	74.29 ± 3.71	66.04 ± 2.98

Table S1 C-index (mean ± SE, 5-fold CV) for all Concat architecture variants using CT-FM embeddings. Columns correspond to modality combinations. **Bold** indicates best performance, underline indicates second best.

The multimodal module $E_m(\cdot)$ introduced in Step 3 consists of two sequential layers, denoted L_1 and L_2. L_1 acts as a bottleneck that projects the concatenated unimodal representations $h_m \in \mathbb{R}^{d_1+d_2+d_3}$ into a reduced-dimensional space (1/3 of the input dimensionality), fusing the three modality-specific vectors into a single representation. L_2 preserves the input-output dimensionality and operates as a non-linear refinement stage before the final fully connected projection to the scalar risk score y. We ablated the architectural composition of E_m by substituting each layer independently with either an FC layer or an ODST block, yielding four configurations: FC+FC, FC+ODST, ODST+FC, and ODST+ODST. Additionally, we evaluated the effect of freezing versus un-freezing the pretrained unimodal encoders during multimodal training. Each configuration was assessed under both encoder settings across all four modality combinations (`CT+Tab`, `WSI+CT`, `WSI+CT+Tab`, `WSI+Tab`). Table S.1 reports C-index values (mean ± SE, 5-fold CV). No single architecture dominates across all modality combinations. FC+FC achieves the highest C-index for `WSI+CT` (76.12, frozen), ODST+ODST peaks on the trimodal configuration (75.29, frozen), and FC+ODST leads on `CT+Tab` (69.90) and `WSI+Tab` (73.30). However, FC+ODST is the only configuration that ranks first or second across all four modality combinations. Regarding encoder optimization, the frozen setting yields the highest or most stable C-index for FC+ODST in three of four combinations, while matching the un-frozen variant on `CT+Tab` (69.90). Freezing the pretrained unimodal modules also preserves the representations learned during Step 2 and reduces the risk of overfitting on the limited cohort. These observations motivated the selection of FC+ODST for the MM with frozen unimodal encoders as the final intermediate fusion strategy, referred as ConcatODST.

S.2 ODST Implementation and Integration

The Oblivious Differentiable Sparsemax Tree (ODST) block [1] is used throughout the proposed framework: as the survival head $E_i(\cdot)$ in each unimodal branch (Step 2) and as the refinement layer L_2 within the multimodal module $E_m(\cdot)$ (Step 3).

Each ODST block implements a differentiable ensemble of oblivious decision trees, in which all nodes at a given depth share the same splitting feature and threshold. Feature selection is governed by learnable logits $\mathbf{f} \in \mathbb{R}^{d_{\text{in}} \times T \times D}$, passed through a `sparsemax` activation [1] that produces a sparse probability vector over input features, summing to one along the feature axis. For each selected feature, the split decision is computed as $(\mathbf{v}_j - \tau) \cdot \exp(-\log \mathcal{T})$, where τ and $\log \mathcal{T}$ are per-node learnable thresholds and log-temperatures, respectively. The resulting logits are mapped to approximate binary routing indicators via `sparsemoid`, a piecewise-linear surrogate of the sigmoid that yields exact zeros outside a bounded interval, enforcing sparse tree routing. Leaf responses are aggregated by computing the element-wise product of bin matches across all D depths, then contracting with a learnable response tensor $\mathbf{R} \in \mathbb{R}^{T \times d_{\text{out}} \times 2^D}$.

Both thresholds and temperatures are initialized in a data-aware fashion. Thresholds are set to random quantiles of the training distribution, sampled from a Beta(β, β) prior with $\beta = 1.0$ (uniform). Temperatures are calibrated so that the majority of data points fall within the linear region of the `sparsemoid` function, controlled by a cutoff parameter set to 1.0. In all experiments, the ODST ensemble comprises T=32 trees with depth D=6 and per-tree output dimension d_{out}=1. All ODST hyperparameters are held constant across modality configurations and fusion strategies.

S.3 Impact of FM Extraction Module on CT Unimodal and Multimodal Modeling

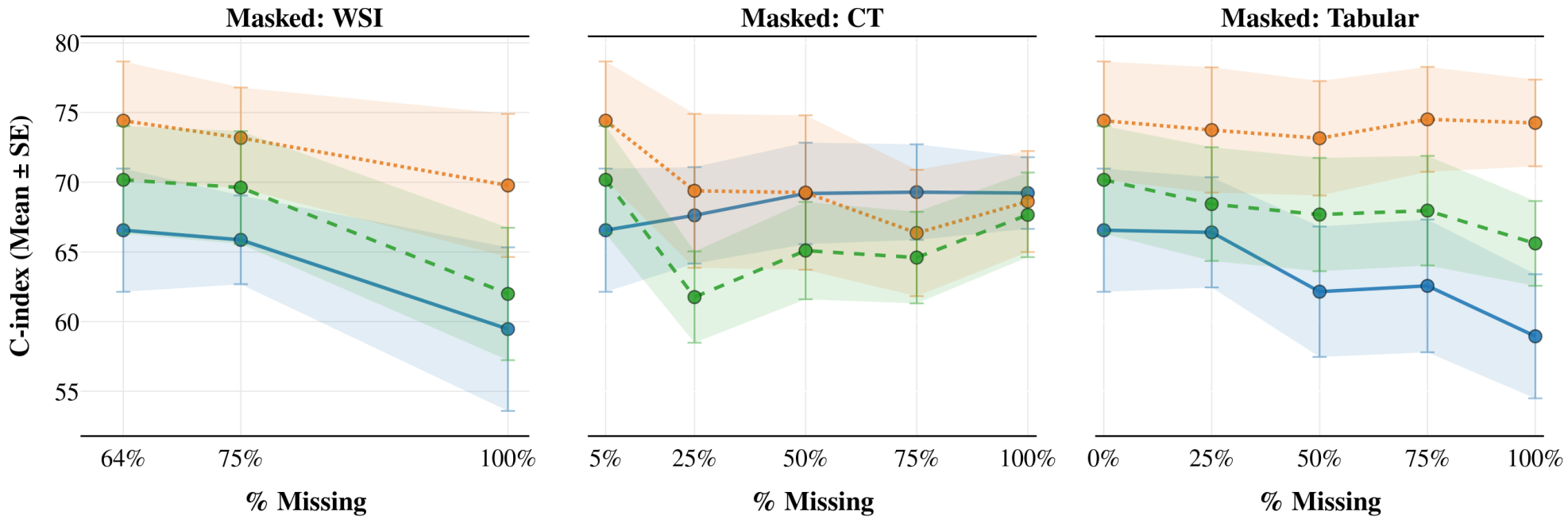

Fig. S1

The choice of foundation model for CT feature extraction has a direct impact on both unimodal and multimodal survival prediction. To quantify this effect, we compared three CT-specific FMs that differ in pretraining strategy and output dimensionality: CT-CLIP [2], a contrastive vision-language model trained on paired CT volumes and radiology reports yielding a 512-dimensional embedding; Merlin [3], a 3D Vision Transformer pretrained via a CLIP-style objective on large-scale CT, report pairs, producing a 2048-dimensional representation; and CT-FM [4], a vision transformer pretrained with masked image modeling on over $148,000$ CT volumes, outputting a 512-dimensional class token. All three FMs encode the full thoracic volume without tumor-specific region selection, isolating the effect of pretraining strategy from input pre-processing choices.

Table S.3 reports unimodal survival performance for all six models across the three CT embeddings. CT-FM yields the strongest results across both ML and DL model families. Among ML baselines, `SGB` trained on CT-FM features reaches a C-index of 70.22 ± 5.05 and Uno C-index of 74.48 ± 5.76, compared to 52.35 ± 1.70 and 51.55 ± 2.37 with Merlin, and 55.62 ± 4.61 and 57.98 ± 5.74 with CT-CLIP. Among DL models, `NAIM+ODST` with CT-FM achieves a C-index of 68.75 ± 5.29, surpassing Merlin (62.20 ± 4.12) and CT-CLIP (56.28 ± 3.72) by 6.55 and 12.47 points, respectively. The training with CT-CLIP embeddings yelded the minimal discriminative signal. `CPH` model reaches a C-index of 50.38 ± 1.91. While Merlin occupies an intermediate position; its ML models (C-index ≤ 52.84) fall below RSF trained on CT-CLIP's embeddings (59.29), yet its `NAIM+ODST` (62.20) exceeds the best CT-CLIP configuration, suggesting that Merlin's higher-dimensional representation ($d = 2048$) benefits from the non-linear interactions captured by the `ODST` layer, while ML models struggle on the larger input space given the limited cohort size.

These differences propagate into multimodal modeling. Figure S1 reports the C-index of the trimodal intermediate fusion model (`ConcatODST`, `WSI+CT+Tab`) under

Modality	Model	Type	C-index	Uno C-index	td-AUC
**CT (Merlin)**	CPH	ML	52.84 ± 2.53	53.51 ± 2.39	58.14 ± 2.34
	RSF	ML	51.21 ± 2.74	53.33 ± 1.71	54.65 ± 3.72
	SGB	ML	52.35 ± 1.70	51.55 ± 2.37	54.39 ± 3.57
	NN	DL	56.98 ± 4.44	57.39 ± 4.64	63.63 ± 6.11
	NAIM	DL	53.69 ± 5.25	54.57 ± 2.06	54.23 ± 3.18
	NAIM+ODST	DL	62.20 ± 4.12	63.17 ± 3.06	64.69 ± 3.87
**CT (CT-CLIP)**	CPH	ML	50.38 ± 1.91	49.90 ± 1.82	49.79 ± 1.36
	RSF	ML	59.29 ± 3.12	62.22 ± 3.20	57.96 ± 3.14
	SGB	ML	55.62 ± 4.61	57.98 ± 5.74	57.91 ± 4.28
	NN	DL	57.41 ± 3.45	61.23 ± 4.82	58.12 ± 3.31
	NAIM	DL	55.14 ± 4.01	58.73 ± 5.42	54.86 ± 3.15
	NAIM+ODST	DL	56.28 ± 3.72	59.96 ± 5.17	56.39 ± 2.98
**CT (CT-FM)**	CPH	ML	58.13 ± 2.51	60.78 ± 2.28	56.88 ± 2.12
	RSF	ML	66.62 ± 6.09	71.90 ± 7.71	61.38 ± 5.15
	SGB	ML	**70.22 ± 5.05**	74.48 ± 5.76	**66.24 ± 4.37**
	NN	DL	59.98 ± 4.24	59.39 ± 3.84	58.63 ± 4.81
	NAIM	DL	63.39 ± 2.25	61.57 ± 2.16	61.23 ± 3.48
	NAIM+ODST	DL	68.75 ± 5.29	**74.57 ± 6.42**	64.42 ± 4.86

Table S2 Unified unimodal performance across WSI, CT and Tabular modalities. Classical ML models (CPH, RSF, **SGB**). DL architectures (NN, NAIM, NAIM+ODST). Mean ± standard error of the mean (SEM, 5-fold CV). The best-performing model for each modality is highlighted in **bold**.

progressive masking of each modality, with volumetric embeddings sourced from each of the three CT FMs. CT-FM (orange) maintains the highest C-index across all three masking conditions and all missingness levels. In the masked WSI panel, CT-FM starts at ≈ 75 and remains above 70 up to complete WSI removal, while CT-CLIP and Merlin drop below 63 and 59 respectively. In the Masked CT panel, CT-FM declines from ≈ 74 at natural missingness (5%) to ≈ 65 at 100%, whereas Merlin and CT-CLIP fall to ≈ 60 and ≈ 63, respectively. In the Masked Tabular panel, CT-FM sustains a C-index above 65 until 75% missing, while Merlin drops below 60 at the same threshold. CT-CLIP tracks between the two across all panels.

Two observations follow from this comparison. First, masking CT in the trimodal model (Figure S1, middle panel) produces a larger absolute drop for CT-FM (from ≈ 74 to ≈ 69) than for Merlin or CT-CLIP, both of which converge to the same endpoint (≈ 69) from lower starting values. CT-FM does not prevent the performance decline upon CT removal; rather, it raises the baseline from which that decline occurs, indicating that its embeddings encode additional prognostic content that the fusion head exploits when CT is available. Second, the Masked Tabular panel reveals a complementary pattern. The gradual removal of the Tabular modality data causes a steep degradation for Merlin (from ≈ 67 to ≈ 58) but has minimal effect on the CT-FM configuration (from ≈ 74 to ≈ 72). This behavior suggests that when the CT backbone provides richer volumetric representations, the fusion model shifts its reliance away

from clinical variables, whereas weaker CT embeddings force the model to depend on Tabular features as the primary prognostic driver. The overall performance gain from CT-FM therefore stems not from CT alone, but from a redistribution of modality importance within the trimodal model that reduces vulnerability to any single data stream.

References

[1] Popov, S., Morozov, S. & Babenko, A. Neural oblivious decision ensembles for deep learning on tabular data. *arXiv preprint arXiv:1909.06312* (2019).

[2] Hamamci, I. E. *et al.* Generalist foundation models from a multimodal dataset for 3d computed tomography. *Nature Biomedical Engineering* 1–19 (2026).

[3] Blankemeier, L. *et al.* Merlin: a computed tomography vision–language foundation model and dataset. *Nature* 1–11 (2026).

[4] Pai, S. *et al.* Vision foundation models for computed tomography. *arXiv preprint arXiv:2501.09001* (2025).

Figure Legends

Figure S1. *Overview of modality availability and feature-level sparsity within the cohort.* Robustness analysis of the intermediate fusion model ConcatODST, under increasing missingness for each modality. Each panel shows the C-index (mean ± SE, 5-fold CV) as a function of the masking rate applied to a single modality while keeping the remaining two at their natural missingness levels (WSI 64%, CT 5%, Tabular 0%). Three CT foundation models are compared as sources of volumetric embeddings: Merlin , CT-FM, and CT-CLIP. In the legend, colors denote the corresponding trimodal configurations with the orange line indicating the setting with the CT-FM embeddings, blue for Merlin, and green corresponds to CT-CLIP.
